# Large-scale preparation of graphene oxide film and its application for electromagnetic interference shielding†

**DOI:** 10.1039/d1ra06070h

**Published:** 2021-10-11

**Authors:** Lijian Xu, Wenqian Zhang, Ledong Wang, Jie Xue, Shifeng Hou

**Affiliations:** School of Chemistry and Chemical Engineering, Shandong University Jinan Shandong 250100 P. R. China; National Engineering Research Center for Colloidal Materials, Shandong University Jinan Shandong 250100 P. R. China; School of Physics, Shandong University Jinan Shandong 250100 P. R. China

## Abstract

In this work, a large-scale preparation of graphene oxide (GO) film is reported, and the structure and the compositional variation was studied after thermal annealing. The electromagnetic interference (EMI) shielding performance of thermally reduced GO films was also investigated. Commercial GO clay was well dispersed by high-speed shearing and formed a stable slurry with a high solid content in water (5%), and this was chosen rather than organic solvent due to its optimal performance in coating procedures and film quality. The optimized thermal annealing procedure resulted in a significant enhancement of electric conductivity and EMI shielding efficiency, which reached 500 S cm^−1^ and 32–42 dB with the thickness under 0.1 mm. The excellent EMI shielding efficiency of thermally reduced GO film, as well as the easily amplified pilot manufactoring procedure adaptive to commercial equipment, produce graphene for universal EMI shielding materials.

## Abstract 2

A large-scale preparation of graphene oxide (GO) film is reported, and annealing process improve the electromagnetic interference shielding performance of the film by structure and compositional variation.

## Graphical abstract

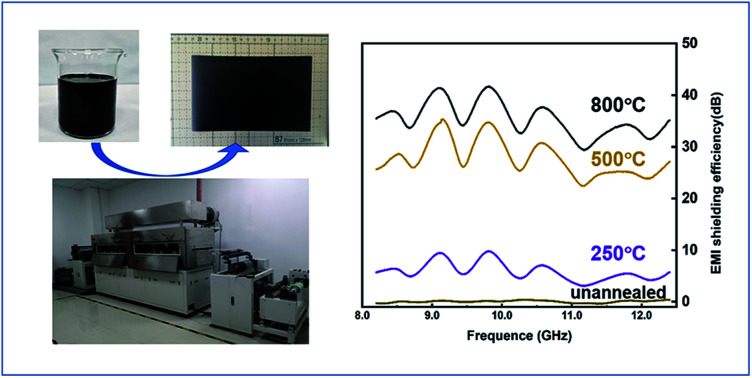

## 1. Introduction

With the rapid development of information technology, excess electromagnetic waves produced by electronic devices and equipment not only cause electromagnetic interference (EMI), which has an impact on the equipment, but it also affects human health.^1–3^ Thus, a series of electromagnetic interference shielding materials has been employed to eliminate the influence of unnecessary electromagnetic waves, which mainly contain metals, conductive fabrics, and carbon materials.^4–7^ Because of the high conductivity of metals, they are satisfactory electromagnetic wave attenuators with high density and low corrosion resistance.^8^ There has also been great interest in carbon materials such as carbon black, graphene, and carbon nanotubes (CNTs) in recent years due to their advantages of being low in density and corrosion-resistant.^9–11^

Graphene has been reported as a promising and effective EMI shielding material because of its excellent electrical properties.^12^ To date, graphene materials with various nano/microstructures or shapes have been built to achieve lightweight and high-performance EMI shielding, including aligned graphene,^13^ three-dimensional (3D) graphene,^14^ graphene sponges,^15,16^ and/or their polymer composites.^17–22^ Most previous studies have focused on the enhancement of EMI shielding efficiency (SE) on a laboratory scale. However, there have been few works describing large-scale fabrication (pilot scale or above) of graphene materials, which impedes their industrial application.

Herein, a large-scale fabrication of a graphene oxide film coating on polymer matrix by a pilot coating machine is reported using high solid content graphene oxide clay manufactured by a modified Hummer's method. The formed stable GO slurry exhibited a more compact film structure and long storage stability. The engineering parameters were preliminarily optimized, and a satisfying batch product was obtained with a variable thickness of 10–150 μm, which can meet the needs of many scenarios. The thermally reduced GO (trGO-1000) film exhibited high conductivity of 500 S cm^−1^ and excellent EMI SE of 45–54 dB (8.2–12.4 GHz, the X-band) with a sample thickness under 0.1 mm. Therefore, the high EMI SE was obtained by an easily amplified annealing process, and together with the pilot fabrication of GO film, can produce graphene with broad prospects for industrial applications.

## 2. Experimental section

### 2.1 Materials

Graphene oxide clay (LNDR-D200J, solid content: 45%) was obtained from LeaderNano Co., Ltd. Polyethylene terephthalate (PET) film was obtained from Tmall.com. Deionized water was produced by a Millipore Elix Essential 5 system. Ethanal and dimethylformamide (DMF) were analytical grade and were purchased from Sinopharm Chemical Reagent Co. Ltd., and were used without further purification.

### 2.2 Preparation of graphene oxide film on the polymer matrix

For the graphene slurry preparation, a certain weight of graphene oxide clay and solvent was mixed in a double planet mixer (HY-DLH7.4L, Guangzhou Hongyun Mixing Equipment Co., Ltd.). A typical mixing procedure was performed for 24 hours under an accelerating mixing speed from 30 rpm to 120 rpm by different stages, with the shearing speed from 1500 rpm to 8000 rpm. Water was necessary to cool the equipment. After the mixing procedure, the slurry settled for 6 hours and was then transferred to the pilot coating machine (MRX-TB300J, Mingruixiang Automation Equipment Co., Ltd.). PET film was chosen as a coating matrix because of its durability and ease of separation. For the entire coating system, the width of the matrix was 300 mm, and the coating width was adjusted to 250 mm; the linear speed was 250 mm min^−1^ with 4 N rolling tension; the drying section operated with 10 L of air volume at 87 °C. The coating thickness of the GO slurry was controlled by the gap between the rollers, typically 1 mm, and 2 mm at maximum. The GO film was manually exfoliated from the PET matrix for further processing.

### 2.3 Thermal reduction of GO films

The tailored GO film was preheated in a 125 °C oven for 24 hours to remove any excess water it contained. A tubular annealing furnace was used for thermal reduction of the graphene oxide film, and the films were pre-cut to fit the furnace. The temperature was increased by stages at 5 °C per minute under 250 °C, over a constant time to a certain peak temperature, which varied from 500 °C to 1000 °C. Four hours elapsed at the peak temperature, and then, the samples were cooled to room temperature at a speed of 10 °C per minute. The annealed samples were marked as trGO-*x*, where *x* represents their peak annealing temperature. Argon was used during all stages as a protective atmosphere.

### 2.4 Characterization

The morphology and strictures of the GO film and trGO film were observed by scanning electron microscopy (SEM, SU8010, Hitachi) and transmission electron microscopy (TEM, JEM-2100, JEOL). The viscosity of the GO slurry was acquired by a rotating viscosity tester (RVDV-1, Shanghai FangRui Instruments). X-ray diffraction (XRD) was performed using an X-ray diffractometer (SmartLab 9 KW, Rigaku) with Cu-Kα radiation (*λ* = 1.54 Å) at a generator voltage of 45 kV. X-ray photoelectron spectroscopy (XPS) was performed using an Al K-Alpha instrument (ESCALAB 250Xi, ThermoFisher). An infrared spectrum was obtained with a Fourier transform infrared spectrometer (Tensor II, Bruker). Thermogravimetric analysis was performed with a thermogravimetric analyzer (TGA 2, Mettler Toledo). The Raman spectrum was obtained with a confocal Raman spectrometer (LabRAM HR800, Horiba). The electrical conductivities were measured using a four-point probe resistivity measurement system (RTS-9, 4 Probes Tech).

### 2.5 EMI performance measurement

Electromagnetic interference shielding efficiency was calculated with microwave scatter parameters (S11, S12, S21, and S22) measured by a vector network analyzer (Ceyear 3672B, CETC) in the X-band (8.2–12.4 GHz) at room temperature. The samples were cut into rectangular sheets with a size of 25.0 × 12.0 mm and sandwiched between waveguide holders. The distance from the sample to port 1 was set as 0, and the length of the sample holder was fixed at 100 mm. The electromagnetic wave had an incident power of 0 dBm, which corresponds to 1 mW. The values of SE total (SE_T_), SE absorption (SE_A_), and SE reflection (SE_R_) were calculated by the following formulas (1)–(6):^23^

1 *R* = |*S*_11_|^2^

2 *T* = |*S*_21_|^2^

3 *A* = 1 − *R* − *T*

4 SE_T_ (dB) = −10 log(*T*)

5 SE_R_ (dB) = −10 log(1 − *R*)

6
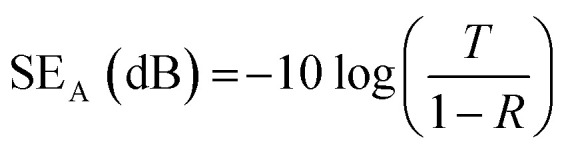
where *R* denotes the reflection coefficient, *T* denotes the transmission coefficient, and *A* denotes the absorption coefficient.

## 3. Results and discussion

### 3.1 Preparation of a GO slurry and its film-forming abilities

**Fig. 1:**
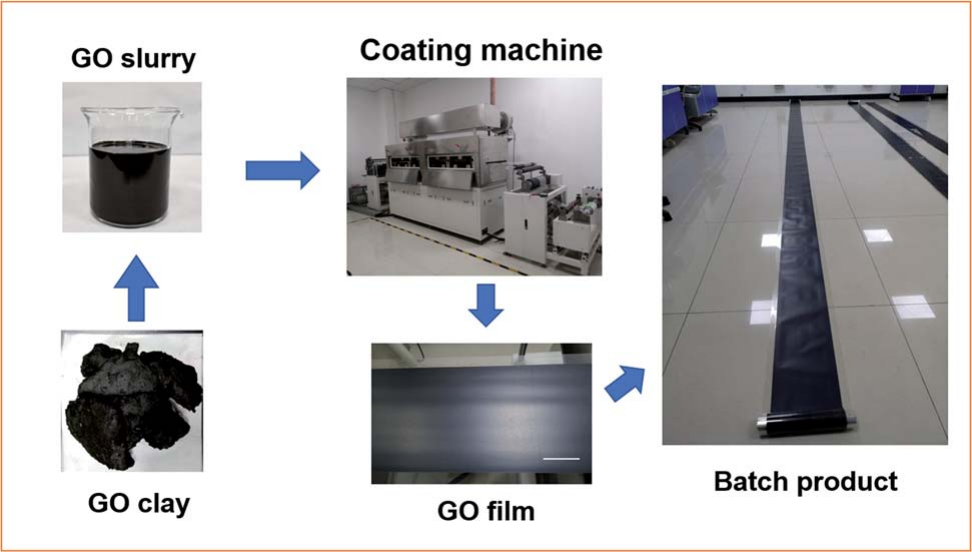
Scheme of large-scale preparation of graphene oxide films.

The process of fabricating graphene oxide film was successfully completed several times, and the scheme is shown in Fig. 1. The graphene oxide slurry formed a flat coating through the gap between the scraper and matrix due to shearing force. Then, external heat was applied to vaporize the solvent in the slurry coating to form the continuous graphene oxide film.

The raw graphene oxide material used in this work was produced by LeaderNano Co., Ltd. in batches. It exhibited satisfactory solubility in water and other hydrophilic solvents because of abundant oxygenic groups on the GO sheet, such as hydroxy and carboxy, which were achieved by a modified Hummer's method. The typical size of the GO sheet was approximately 2 μm, and it was semi-transparent at high magnification as a representation of a single-layer material. Slight folding occurred, which was due to the evaporation of water during the TEM sample preparation, and the intrinsic morphology of the graphene sheets should appear as fully stretched after a long period of high-speed shearing.

**Fig. 2:**
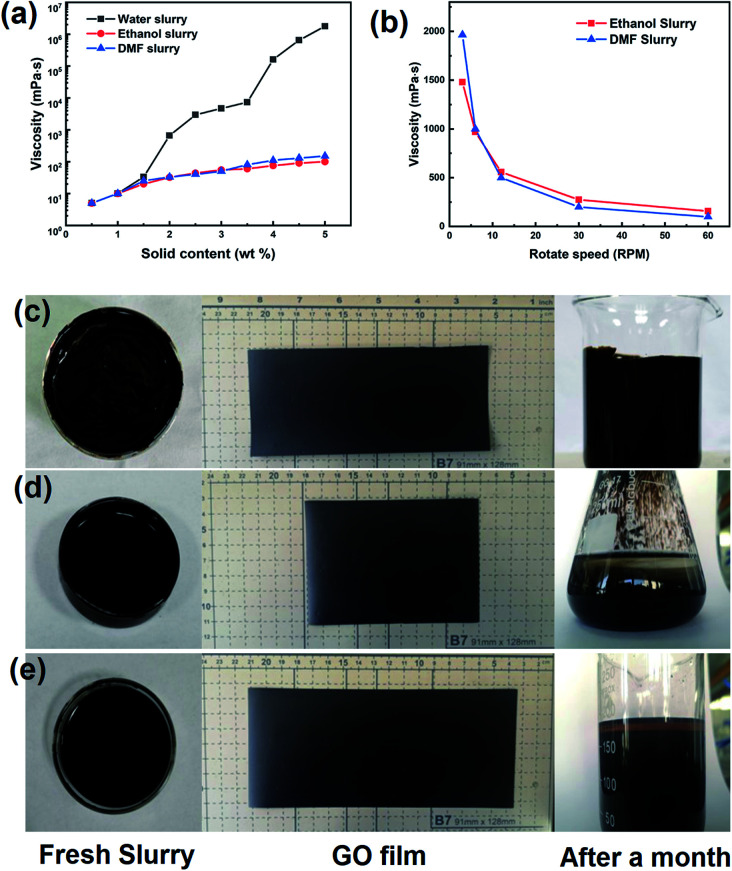
Properties of GO slurry. (a) Viscosity curves of different slurry; (b) rheology feature of organic GO slurry. The appearance of fresh slurry, dry-film and long-storage slurry made of water (c), ethanol (d) and DMF (e).

For the pilot coating procedure, the amount of solids in the slurry is a key to the film formation because of the conservation of mass. The ratio of the GO film thickness to slurry thickness was nearly equal to the solid content of the slurry. From one-pot moulding to final application, a higher solid content should be more optimal. Viscosity is an important process parameter associated with solid content, and it demonstrates the interactions between solvent and solute. The viscosities of a GO slurry made with water, ethanol, or DMF were investigated by a rotating viscosity tester, and the results are shown in Fig. 2a.

The results clearly differed according to solvent, where the viscosity of the water-based slurry was much higher than that of the organic solvent-based slurries with the same amount of solid. The viscosity of the water-based slurry exponentially increased with a coefficient of approximately 1 by increasing the amount of solid. The slurry showed a viscosity of 7300 mPa s@3.5 wt% and 650 000 mPa s@4.5 wt%. The organic solvent-based slurries have very low viscosity (under 300 mPa s@5.0 wt%) and little variation among the testing range relative to the water-based slurry. This may be attributed to the high hydrogen bond density in water. These intermolecular interactions enhanced the attraction between GO sheets and work against the shearing deformation.

Hydrogen bonds are a weak interaction, and they are easily broken by strong external shearing force and are rebuilt when the situation returns to stability. All types of slurries showed non-Newtonian fluid characteristics similar to those of polymer. The viscosity curves of organic solvent-based slurry varied with the rotation speed, and are shown as Fig. 2b. The organic solvent-based slurries both contained 5.0% solid content, while their apparent viscosities were approximately 100 mPa s@60 rpm. When the rotation speed was reduced to 3 rpm, the apparent viscosities increased to approximately 2000 mPa s. This rheological feature of a GO slurry limits the coating process because the heading speed of the matrix was in a fixed interval. In fact, PET was chosen as a matrix because of its robust mechanical variation, and Al foil and Cu foil failed in a pre-coating test as a preparation for a high-viscosity slurry. As a compromise to the pilot equipment, the requirement of the slurry viscosity was set as 10 000 mPa s, which resulted in a stable thick slurry coating on the PET matrix.

The film-forming ability is the main indicator used to test the adequacy of a coating slurry. All the different types of slurries had finished the pre-coating test conducted on a small auto-coating machine (MRX-TMH250, Mingruixiang Automation Equipment), and the thickness of the slurry coating was set as 1 mm on the PET matrix. The amount of solid in the organic solvent-based slurry was adjusted to a proper viscosity that would form a stable slurry coating. The coated samples were transferred to an oven maintained at 40 °C until fully dried.

The appearances of the slurries and films are shown in Fig. 2c–e. The gross view of the GO films reveals no significant difference in surface flatness or roughness. The static appearances of different fresh GO slurries were nearly the same, but for long-term storage, the precipitation of the GO sheets became obvious in the organic solvent-based slurries. However, this did not occur with the water-based slurry.

**Fig. 3:**
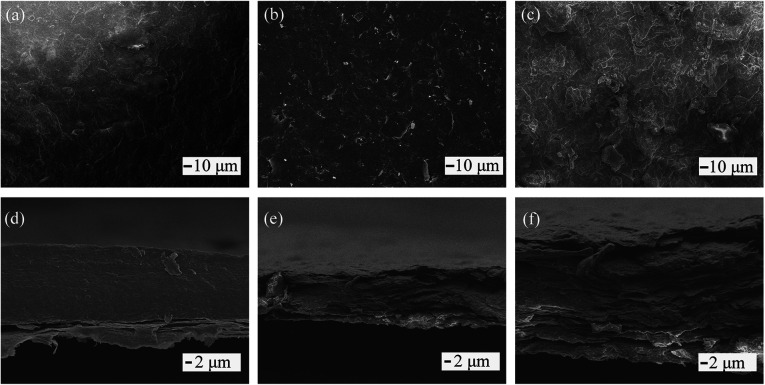
(a, b, and c) Top view and (d, e and f) section view of GO films made by water slurry (a and d), ethanol slurry (b and d), and DMF slurry (c and f).

SEM images show that the film composed of a water-based slurry was approximately 50 μm in thickness, which is similar to the theoretical value, and exhibited a compact sectional structure without cavities or defects. There were a few defects observed on the film surface, as shown in Fig. 3, and the magnified image (Fig. 3a and d) shows a smooth GO sheet with some small wrinkles. The GO sheets were well spread in water by their satisfactory hydrophilicity, and a tight arrangement was obtained due to the tension caused by evaporation of interlayer water. For films made of an organic solvent-based slurry, additional defects such as holes and upwarp were observed on the film surface (Fig. 3b and c) and section (Fig. 3e and f), which may be due to the incomplete extension or curling of the GO sheets. Additionally, the lower surface tension and rapid evaporation of the organic solvent may also contribute.

Using water as a solvent is suitable for both the slurry-coating process and material storage, and also does not contribute to environmental pollution caused by organic solvent vapor. Therefore, the GO films used below are all made from a water-based slurry (1 cm GO slurry coating with 5% solid content) with a thickness of 50 μm and without any other modifications.

### 3.2 The structure and properties of GO film and trGO films

**Fig. 4:**
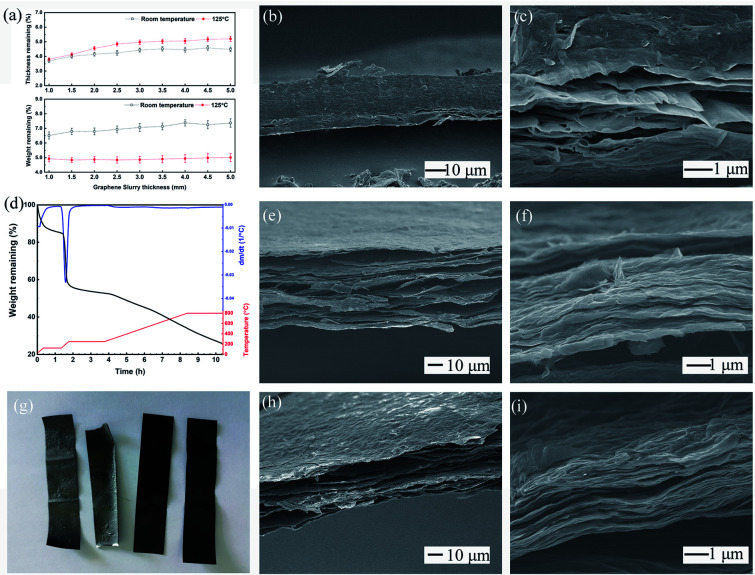
(a) The variation of thickness and weight after preheating; (b and c) the section view of pre-heated GO film; (d) TGA curve of trGO-800; (e and f) the section view of trGO-250; (g) the appearance of trGO-800, trGO-250, preheated GO and raw GO film; (h and i) the section view of trGO-800.

The thermal treatment process was divided into two parts, and the temperature curve was optimized in several stages in this work. The first pre-heating at 125 °C lasted for 24 hours to remove the excess water contained inside the GO films. This process was established by the feedback from subsequent testing results, and could be avoided by increasing the size of the production line *via* extending the drying section with gradual increases in temperature. The thicker the GO films, the more water contained inside them. Fig. 4a shows that pre-heating removed most of the water inside the GO films. Interestingly, all the samples tested were produced by a water slurry with a solid content of 5%. The thin slurry coating showed greater offset on the film thickness to the theoretical value, while the weight remaining ratio was kept consistent. This may have occurred because of the compact stack of the graphene sheet driven by the surface tension of the interlayer water during the evaporation process.

The sectional morphology of a preheated sample is shown as Fig. 4b and c. The total film structure was relatively intact, with small interlayer cracking that occurred near the film surface, and the surface sheets curled off the films. A magnified image of the cracking zone shows that most stacked sheets were not totally separated, but formed small tunnels with a diameter of nanometers that can act as an exit for interlayer water molecules. The separated sheets in Fig. 4c exhibit small wrinkles, which indicates that the preheating temperature was near the critical zone, and the film would expand under higher treatment temperature. The optimal length and temperature of this period are open to debate, but from the results of subsequent experiments, our scheme is feasible. After the pre-heating, the GO film samples were cut into small sizes pieces for further annealing processes.

The TGA curve shows the variation in the weight of samples during the preparation of trGO-800 made with fresh pilot product, as illustrated in Fig. 4d. The first stage for 1 hour stabilization at 125 °C was set to smooth any possible tension caused by the thermal history (during the drying section) and remove the excess interlayer water in accordance with the previously mentioned procedure. The sample's weight clearly decreased during this stage and showed a weight loss of 13%, which approximated the results shown in Fig. 4a. During the second stage, the temperature was increased to 250 °C by 5 °C per minute, and was maintained for 2 hours. The sample lost more than 30% of its initial weight during this stage, and showed a dramatic decrease above 200 °C, which was mostly due to the complete removal of the water contained as interlayer water and bound water.

The cracking of the oxygenic group on the graphene sheets also contributes, as the elemental analysis results show in Table 1. After stabilization at 250 °C, a fixed period (4 hours) elapsed with the goal of reaching the peak temperature. This was maintained for 2 hours so that the samples would be fully annealed, and this exhibited a smooth weight loss with a linear relationship with time as opposed to the decomposition of carboxyl on the interior GO sheets.

**Table 1:** The element analysis and conductivity of raw GO and trGO samples

Sample	Conductivity (S cm^−1^)	Element content (%)				
		N	C	H	S	O
Raw GO	—	0.02	47.17	3.028	0.32	49.46
trGO-250	0.001^a^	0.00	74.94	0.95	0.12	23.99
trGO-400	0.16	0.00	77.17	1.03	0.43	21.36
trGO-600	10	0.01	95.58	1.19	0.22	3.01
trGO-800	500	0.02	97.23	1.23	0.15	1.48

a The lower limit of testing instrument.

The gross view of the samples before and after thermal treatment is shown as Fig. 4g. The as-prepared GO film (1st from right) was pre-heated for 24 hours@125 °C (2nd from right), and exhibits an approximate appearance with no obvious bending or curling. The trGO-250 (3rd from right) exhibits a grey color and graphite shine, which indicates that graphitization of the surface sheets of the film had occurred. Some bending at the ribbon edges and some small humps were found on the surfaces, and were possibly caused by the asymmetrical internal stress that arose from group decomposition. The trGO-800 (4th from right) exhibited additional humps and bending among the ribbon, and the shine and texture was the same as that of trGO-250, which demonstrated that the completeness of graphitization cannot be determined by the appearance of the annealed films.

SEM images of trGO-250 and trGO-800 are shown in Fig. 4. The total section views of the sample show obvious differences around the interlayer gap. The section of trGO-250 (Fig. 4e and f) exhibits the impact stacking structure and shows a more parallel gap uniformly distributed among the layers. The magnified image of section part (Fig. 4f) shows more wrinkles on the sheets as compared to the preheated sample (Fig. 4c), and the trace of a tunnel could be observed and connected to form the interlayer gap. For the trGO-800 (Fig. 4h and i), the stacking structure was nearly destroyed by the decomposition of the sheet, and showed a foam-like appearance with irregular holes inside the film. The surface layers of trGO-800 (Fig. 4h) show small fluctuations spread all over the plane, and crevices between single sheets were also found on the surface, which depicts the separation of the stacked sheets. The magnified image of trGO-800 (Fig. 4i) shows additional wrinkles on sheets and larger gaps between the layers, which is coincident with the thermal expansion of graphene material. The expansion of the film resulted in an increase in film thickness (50 μm to 65 μm by trGO-800), which is larger than that of the sample treated at lower temperature. The samples treated at higher temperature exhibited the same micro-morphology as that of trGO-800. For large-scale preparation of the trGO film, the irregular transformation of trGO film relative to flat GO film would hamper its application. A few trials were performed to solve this problem, but there has not been much progress.

**Fig. 5:**
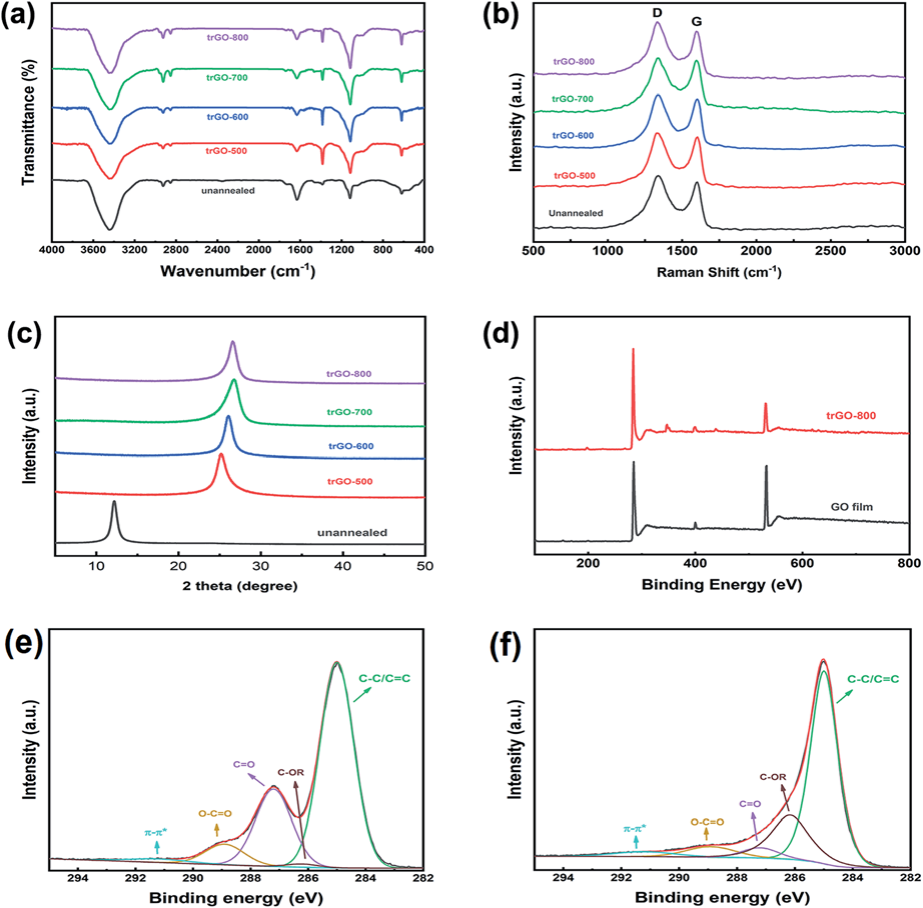
(a) FTIR spectra, (b) Raman spectra and (c) XRD pattern of raw GO and trGO samples; (d) total XPS curves of raw GO film and trGO-800; and high solution C 1s spectra of GO film (e) and trGO-800 (f).

FTIR was employed to study the variation in the chemical groups on the GO sheets during the annealing process, and the results are shown as Fig. 5a. The spectra of unannealed GO film exhibited several peaks of typical oxygenic groups on the GO sheets, such as –OH (3446 cm^−1^), –COOH (1630 cm^−1^), and –C–O–C– (1104 cm^−1^). After the thermal annealing process, all the trGO samples underwent considerable weight loss by the decomposition of oxygenic group, which actually was the carboxy, because the peak located at wavenumber 1630 cm^−1^ obviously decreased, while there was little change in the broad peak at 3440 cm^−1^. As a consequence of decarboxylation, C–H bending was observed at 1385 cm^−1^ on the spectra of trGO samples, and the peak at 2925 cm^−1^ represents the formation of a methylene structure.^24^ With the increase in annealing temperature, the –OH became decomposed, and the –C–O–C was generated as another type of deoxidization. For trGO-500, the C–H content was obviously higher than that of other trGO samples, which was supposed to be intermediate amorphous carbon unstably attached to the graphene sheets. This peak decreased with the increase in annealing temperature due to the instability of non-aromatic carbon. It was concluded that the annealing process in our work removed most oxygenic groups but could not reduce the oxygen content to zero, and these data are also compatible with the results of the element analysis.

To investigate the effect on the integrity of the sheets during the annealing process, a Raman spectrometer was applied to detect the defects in the trGO sheets (Fig. 5b). The unannealed GO film exhibited a typical feature consisting of a double-peak spectra with a D band at 1350 cm^−1^ and a G band at 1590 cm^−1^. The oxidation of graphite caused structural defects and a disordered configuration expressed as the D band, and the G band represented the entire graphitic structure. The annealing process in our work resulted in the removal of additional oxygenic groups, but it was incapable of rebuilding the graphitic structure, which should be performed above 2800 °C. The intensity ratio of the D band to G band was similar (*I*_D_ : *I*_G_ = 1.19 by raw GO, and 1.13 by trGO-800), which showcased the amount of ordered sp^2^ and disordered sp^3^ carbon domain changes that occurred as a result of the decomposition of carboxyl groups added to the graphene sheets.

Fig. 5c displays the XRD pattern of unannealed GO film and trGO films treated under different peak temperatures. A single strong peak located at 2*θ* = 12.2° of the unannealed GO and no peak at 2*θ* = 26.5° (002 of graphite) proved that the sheets in the raw graphene oxide clay were fully separated by the oxidation of graphite. The gap between the graphene oxide sheets did not decrease during the slurry preparation and coating process. After thermal annealing, the XRD pattens of the trGO samples showed a significant shift near 2*θ* = 26.5°, which was caused by the decrease in the interlayer space following the decomposition of the oxygenic groups on the graphene sheets. The trGO-500 exhibited a peak located at 2*θ* = 25.2° and for trGO-600, at 2*θ* = 26.0° while the peaks of trGO-700 and trGO-800 were both located at 2*θ* = 26.5°. These data also indicate that the additional group obtained by graphite oxidation could be mostly removed by the annealing process above 700 °C, which is coincident with the results above.

In the XPS analysis of unannealed GO film and trGO-800, shown in Fig. 5d, which was used to ensure that chemical composition changes occurred during the annealing process, the relative intensity of O 1s (approximately 532 eV) to C 1s (approximately 285 eV) decreased after the annealing process. This clearly implies that thermal reduction occurred during the annealing process, and some oxygenic groups were removed. According to the FTIR results and previous studies, it was confirmed that there are different carbon components, including C–C/C=C (approximately 285.0 eV), C–OR (approximately 285.4 eV), C=O (approximately 287.4 eV), O–C=O (approximately 288.9 eV), and π–π* (291.7 eV). The XPS spectra of the C 1s region of unannealed GO film (Fig. 5e) and trGO-800 (Fig. 5f) illustrate this more intuitively. The spectra show that the C=O content of trGO-800 was greatly decreased compared to raw GO, which indicates that decarboxylation occurred.

### 3.3 The conductivity and EMI shielding performance of trGO films

Conductivity of the annealed films was tested using four-point probe resistivity measurements. The results in Table 1 show that the annealing process has a great influence on the electrical conductivity because a portion of oxygenic groups are removed, and the hexagonal structure becomes more suitable for the migration of the current carrier. The initial GO film shows high resistivity and nonviability for the four-probe method. After thermal reduction, an efficient conductive network began to be constructed in the trGO samples, and the higher temperature at which the films annealed, the greater the conductivity enhancement by the films. The highest conductivity of 500 S cm^−1^ occurred when the annealed temperature was above 800 °C, which was much larger than what was achieved by the other samples.

**Fig. 6:**
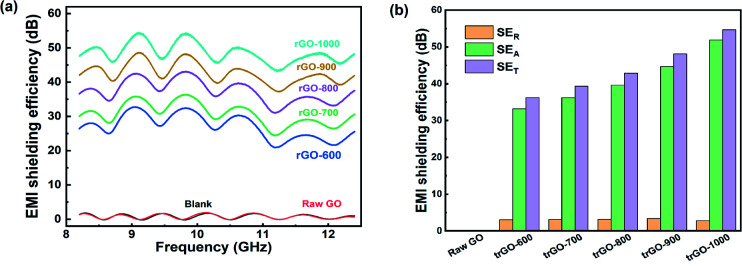
(a) The total EMI shielding performance of trGO samples and raw GO film; (b) SE_T_, SE_A_ and SE_R_ of trGO samples.

The EMI shielding performance was investigated by testing the samples with a vector network analyzer at a frequency range of 8.2–12.4 GHz, as represented in Fig. 6. All samples exhibited a mild floating electromagnetic shielding performance among the X-band, and the appearance of the EMI SE curves was the same, with several peaks above the satisfying baseline. The maximum EMI SE of each sample appears with frequencies of 9.3 GHz and 9.8 GHz. With the increase in the annealing temperature, the EMI SE is dramatically increased, which illustrates the significant relativity between conductivity and EMI SE.

As the treatment temperature increased above 500 °C, the EMI SE of the thermally reduced graphene films exceeded the commercial requirement standard value of 20 dB. The maximum EMI SE of trGO-1000 is as high as 54 dB@9.8 GHz, which is superior to values in the reported literature. Further investigation revealed an obvious tendency of variation in each SE component, as shown in Fig. 6b. As observed in Fig. 4, the trGO sample did not undergo a large increase in thickness from the initial GO film, but many cavities were formed among the layered structures that could increase the SE_A_ by internal multiple reflections.

The sheets of trGO-800 were fully separated by the thermal treatment, which indicates that additional interfaces were created to enlarge the means by which multiple reflections occur. Additionally, the increased conductivity resulted in a greater mismatch of microwave impedance between material and air, and also resulted in interface reflection, which may explain the enhancement of SE_R_ for the trGO samples.^25,26^

## 4. Conclusions

We demonstrated a pilot process of graphene oxide film production and its application as an EMI material. Commercial graphene oxide clay was used with water as the solvent for the slurry, which was compatible with a conventional coating machine and yielded GO films with varied thickness from 10 μm to 100 μm. This procedure could be easily amplified to obtain batch products. The thermal reduction of the GO film removed the oxygenic groups on the graphene sheet and increased the conductivity of the film without destroying its integrity. The trGO sample exhibited enhanced conductivity and EMI performance with an increased annealing temperature.

The most optimal annealing temperature for samples with 50 μm thickness was 1000 °C in our work. The trGO-1000 exhibited electrical conductivity as 500 S cm^−1^ EMI shielding efficiency above 45 dB among the X-band and a maximum of 54.3 dB@9.8 GHz. For the excellent performances and operability of the batch product, the trGO film reveals great potential in high efficiency EMI shield applications.

## Conflicts of interest

There are no conflicts to declare.

## Supplementary Material

RA-011-D1RA06070H-s001
